# Salivary inflammatory biomarkers and glycated haemoglobin among patients with type 2 diabetic mellitus

**DOI:** 10.1186/s12903-021-01453-y

**Published:** 2021-03-06

**Authors:** Ekhosuehi Theophilus Agho, Foluso John Owotade, Babatope Ayodeji Kolawole, Elijah Olufemi Oyetola, Tewogbade Adeoye Adedeji

**Affiliations:** 1 Department of Dental Surgery, Ahmadu Bello University, Zaria, 810107 Nigeria; 2 Department of Oral Medicine and Oral Pathology, Obafemi Awolowo University, Ile-Ife, 220005 Nigeria; 3 Department of Medicine, Obafemi Awolowo University, Ile-Ife, 220005 Nigeria; 4 Department of Chemical Pathology, Obafemi Awolowo University, Ile-Ife, 220005 Nigeria

**Keywords:** Type 2 diabetes mellitus, Saliva, Inflammatory biomarker, Glycated haemogblobin

## Abstract

**Background:**

Type 2 diabetes mellitus has reached epidemic proportions worldwide and improved detection techniques and biomarkers are urgently needed across the spectrum of diabetes initiation and progression. Inflammatory biomarkers play a role in the development of the condition and blood is the gold standard body fluid for the diagnosis of diabetes mellitus. Serum glycated haemoglobin is a widely used marker of chronic hyperglycemia, and it is currently used to diagnose type 2 diabetes mellitus and it is the standard biomarker for the adequacy of management. However, saliva offers an alternative to serum as a biological fluid for diagnostic purposes. Non-invasive measures of inflammatory biomarkers (such as saliva diagnostics) are increasingly being investigated due to significant similarities between salivary and serum proteome. The role of saliva diagnostics in diabetes mellitus has not been explored in our study population.

**Objectives:**

This study investigated the association of selected salivary inflammatory biomarkers (Interleukin 6 [IL-6], C-reactive protein [CRP], and Tumour necrosis factor α [TNF-α]) to glycated haemoglobin (HbA1C) in type 2 diabetics.

**Materials and methods:**

Seventy-five participants, 39 type 2 diabetics (52%) and 36 (48%) healthy controls were recruited. Saliva and blood samples were collected for each participant. The levels of selected salivary inflammatory biomarkers (IL-6, CRP and TNF-α) were estimated by Enzyme Linked Immunosorbent Assay (ELISA) method and glycated haemogloin (HbA1C) was estimated using the liquid chromatography method. Periodontal status of the participants were determined using the Basic Periodontal Examination (BPE).

**Results:**

The mean salivary levels of CRP was significantly higher in diabetics, 0.05 ± 0.04 µg/ml than in controls, 0.02 ± 0.02 µg/ml (p < 0.001). Mean TNF-α was also significantly higher in diabetics, 5.39 ± 12.10 pg/ml than in controls, 1.51 ± 3.66 pg/ml (p = 0.036). Mean salivary IL-6 was also higher in diabetics compared with controls (47.20 ± 18.49 versus 41.94 ± 16.88 pg/ml), but the difference was not statistically significant, p = 0.204. In the multivariate analysis adjusting for age and periodontal status, only the mean salivary CRP was significantly higher in diabetics, 0.034 higher than controls (95% CI 0.009, 0.059 and p = 0.01). There was a positive correlation between salivary CRP and HbA1C levels, which was moderate with r-value 0.4929 and p-value < 0.0001.

**Conclusions:**

Salivary inflammatory biomarkers especially CRP are higher in diabetics compared with controls and CRP is positively correlated with serum HbA1C levels. The biomarkers show potentials as non-invasive alternative method to evaluate glycaemic control in diabetes.

## Introduction

Diabetes mellitus is a group of metabolic diseases characterized by hyperglycaemia resulting from defects in insulin secretion, insulin action or both [1]. The prevalence of diabetes mellitus is increasing globally, as the number is expected to rise to 300 million by 2025 and 366 million by the year 2030 [2]. In Nigeria, about 5 million people are currently living with diabetes mellitus, with more than 1.56 million cases recorded in 2015 alone [3]. The prevalence of diabetes mellitus in Nigeria ranges from 0.56% in the rural areas and up to 11% in urban Lagos [4], with a mean prevalence of 1.9% [3].

Currently, diabetes mellitus is diagnosed by evaluating blood glucose level, however, monitoring glycated haemoglobin levels is a frequently and relatively accurate measure of average glycaemic control [5]. Monitoring of biochemical parameters of diabetes mellitus such as fasting blood glucose levels and glycated haemoglobin levels, typically involves invasive techniques associated with pain and distress [6]. The development of non-invasive methods for frequent sampling of biomarkers is a growing area of research. Salivary biomarkers indicate the existence or the risk of developing a disease, as well as the response to a particular therapy [7]. Furthermore, saliva offers significant potential as diagnostic tool for the surveillance of disease due to its similar proteomics with blood [8] and it is an inexpensive, simple and non-invasive screening method. Salivary diagnostics is a rapidly emerging field that is dependent on the development of sensitive and specific biomarkers that can be employed in large scale clinical setting. This is particularly important in sub Saharan Africa where undiagnosed diabetes mellitus range from 40 to 100% [9]. Even when diagnosed, monitoring is inadequate owing to insufficient laboratory facilities [10]. The ability to employ a simple analysis of body fluid such as saliva for rapid, non-invasive testing of validated novel analyte will present a substantial protocol for the diagnosis and management of diabetes both locally and globally.The development of simple non invasive options such as saliva testing can make a difference.

The aim of this study was to determine and compare selected salivary inflammatory biomarkers with serum glycated haemoglobin in Type 2 diabetics and healthy controls.

Our study hypothesis is that the saliva inflammatory biomarkers will be higher in Type 2 diabetics and our study investigated the association between salivary levels of selected salivary inflammatory biomarkers (CRP, IL-6 and TNFα) in type 2 diabetes mellitus and glycaemic control. The outcome may contribute to knowledge on developing standardized protocol that uses saliva as a diagnostic tool for screening, monitoring and management of diabetes mellitus.

## Materials and methods

### Study design

Case control study was performed on type 2 diabetics attending the Diabetes clinic of the Obafemi Awolowo University Teaching Hospitals’ Complex Ile-Ife, Osun State, Nigeria. Non diabetic patients attending the Dental Outpatient clinic of the hospital that met the inclusion criteria were recruited as controls. The study was conducted over a period of six months. From March 1st–August 31st 2018. Ethical approval was obtained from the Ethics and Research Committee of the Obafemi Awolowo University Teaching Hospitals Complex, Ile- Ife (IRB/IEC/0004553 and NHREC/27/02/2009a). Written informed consent was obtained from all participants during the study.

### Sample size estimation

The minimum sample size required for this study was estimated using the formula that compares two means. The parameters used for estimation were derived from a previous study. The mean value of IL-6 in the saliva of type 2 diabetes mellitus patients is 69.3 and 53 for healthy controls with a pooled standard deviation of 18.6 [6]. With a power of 90% and alpha of 5%, a sample size of 67.7 was obtained. Seventy five participants were recruited, 36 type 2 diabetics and 39 apparently healthy controls.

### Selection criteria

All type 2 diabetic consenting patients with a score of 5 or higher of the diabetes risk test at the time of diagnosis as proposed by the American Diabetes Association 2017 [11] and aged 20 to 70 years were recruited. Diabetes risk test:

1. How old are you? ……….
  Less than 40 years (0 points)
  Less than 40 years (0 points)
  40–49 years (1 point)
  50–59 years (2 points)
  60 years or older (3 points)
2. Are you a man or woman?
  Man (1 point) Woman (0 points)
3. If you are woman, have you ever been diagnosed with gestational diabetes?
  Yes 1(point) No (0 points)
4. Do you have a mother, father, sister or brother with diabetes?
  Yes 1(point) No (0 point)
5. Have you ever been diagnosed with high blood pressure?
  Yes 1(point) No (0 point)
6. Are you physically active
  Yes (1 point) No (0 points)
7. What is your weight category?

Participants with other forms of diabetes mellitus besides type 2 with history of salivary gland surgery who were either pregnant or smokers or with any other known systemic diseases were excluded.

Consenting healthy controls with no known history of diabetes mellitus, other systemic diseases, who were not on any drug therapy, who were neither smokers nor pregnant and aged 20 to 70 years were also recruited.

### Collection of samples

Before saliva and blood sample collection, participants were asked to rinse out their mouths with water to remove food debris, sit still in a comfortable position for six minutes and were not allowed to chew or speak until the saliva sample was collected. Unstimulated whole saliva was collected in the morning between the 8.00am and 11.00am from type 2 diabetics and healthy controls using the spit method every sixty seconds for 5 min. The saliva samples, stored in a sterile plain bottle were immediately taken to the laboratory in an ice pack at 4 °C and then frozen at or below—20 °C as immediately. Samples were defrosted at room temperature and then centrifuged at 6000 rpm for 10 min to remove contaminants such as oral epithelial cells, microorganisms and food debris. Venepuncture was done at the cubital fossa to collect blood sample from participants under sterile procedure using 5 ml syringe and stored in EDTA bottle.

### Assessment of periodontal status

Basic periodontal examination (BPE) a simple and rapid screening tool used to indicate the level of examination needed and to provide basic guidance on treatment need was used to assess the periodontal status. [12] A World health Organization (WHO) probe was used for this examination. This has ‘ball end’ 0.5 mm in diameter and a black band from 3.5 to 5.5 mm. Light probing force was used (20–25 g). The probe was walked round the teeth in each sextant. All sites were examined to ensure that the highest score was recorded before moving to the next. The participants were categorized using the scoring codes as:

- Code 0: Given to the sextant if there were no pockets exceeding 3.5 mm (black band entirely visible), no calculus or overhangs of fillings, no bleeding on gentle probing.
- Code 1: Given to the sextant if there were no pocket exceeding 3.5 mm (black band entirely visible), no calculus or overhang of fillings but bleeding on gentle probing.
- Code 2: Given to the sextant if there were no pocket exceeding 3.5 mm (black band entirely visible) but calculus and plaque retention factors are seen at or recognized underneath the gingival margin.
- Code 3: Given to the sextant if the colour-coded area of the probe remains partially visible when inserted when inserted into the deepest pocket indicating pocket depth between 3.5 and 5.5 mm
- Code 4: Given to a sextant if at one or more teeth, the colour-coded are of the WHO probe disappears into the pocket indicating a pocket of 6 mm or more.
- Code *: Pocket + recession (CAL) at least 7 mm in total; Furcation involvement

Only the highest score was recorded for each sextant.

Periodontal evaluation, comprising of pocket depth (PD) and simplified oral hygiene score (OHI-S) were evaluated. Participants with ≤ 15 teeth, BPE score of 4 were excluded from the study.

#### Estimation of salivary CRP

The salivary CRP levels was assessed using the ELISA test kit (Monobind Inc. Lake Forest, CA 52,630 USA). Before proceeding with the assay, all reagents, serum references and control were brought to room temperature (20–27 °C).

#### Estimation of salivary IL-6

The saliva levels of IL-6 was assessed using the ELISA test kit (AVISCERA BIOSCIENCE INC, USA). All reagents and sample were brought to room temperature before use.

#### Estimation of saliva TNF-α

The levels of TNF-α in saliva was estimated using ELISA kit (AVISCERA BIOSCIENCE, INC, USA). All reagents and sample were brought to room temperature before use.

### Estimation of serum glycated haemoglobin (HbA1C)

Commercial glycated haemoglobin kit (BIO QUANT DIAGNOSTICS) was used.

### Statistical analysis

Data entry and analysis was done using STATA 14 statistical software (Statacorp, College station, Texas USA). Descriptive statistics, bivariate analysis such as paired t- test for IL-6 and Mann–Whitney U test were used as appropriate to compare the two groups. Spearman correlation coefficient was used to determine the relationship between salivary inflammatory biomarkers and serum levels of HbA1C in subjects with type 2 diabetes. Confidence level was set at 95% and p value of ≤ 0.05 was considered significant. Multivariate regression model was using the biomarkers CRP, IL-6 and TNF-α as outcomes and diabetic state, periodontal status and age as predictors. CRP and TNF-α were slightly skewed and log transformed, however the conclusion was not different without transformation and were fitted without transformation. Standard techniques were used for model checking.

## Results

Seventy five participants, 39 (52%) diabetics and 36 (48%) healthy controls completed the study. Forty-three participants (57.4%) were males while 32 (42.6%) were females. The age range of the participants was 20–70 years and mean age standard deviation (SD) was 46.2 (15.4). Diabetics were older with a mean age of 57(12) years compared with 34.5(8.6) years in the controls (p < 0.01) Majority of the diabetics were in the age bracket/group between 40 to 59 years. The mean (SD) body mass index BMI were 26.1 (8.2) kg/m^2^ and 25.9 (5.3) kg/m^2^ for healthy controls and diabetics respectively. Furthermore, periodontal status assessed using Basic Periodontal Examination (BPE) revealed that none of the diabetics had healthy periodontium (BPE code 0) and none had overt periodontitis (BPE code 4). Majority of the diabetics, 31 (79.5%) had BPE code 2 (p 0.51) (Table 1).

**Table 1:** Sociodemographic characteristics of the participants

Variables	Controls (N = 36)	Diabetics (N = 39)	Total (N = 75)	p value
Gender	Frequency (48%)	Frequency (52%)	Frequency. (100%)	
Male	26 (34.7%)	17 (22.7%)	43 (57.4%)	**0.012**
Female	10 (13.3%)	22 (29.3%)	32 (42.6%)	
Age		2		
20–29	10	3		
30–39	15	11		< 0.01*
40–49	9	15		
50–59	2	5		
60–69	–	3		
70	–	57.0 (12.0)	46.2 (15.4)	
Mean age (SD) years	34.5 (8.6)			
Marital status				
Single		2 (2.7%)	18 (24.0%)	
Married	16 (21.3%)	34 (45.3%)	53 (70.7%)	**0.51**
Divorced	19 (25.3%)	1 (1.3%)	1 (1.3%)	
Widow/widower	0 (0%)	2 (2.7%)	3 (4.0%)	
Body mass index (SD) kg/m^2^	1 (1.3%)			
Periodontal status (BPE)	26.1 (8.2)	25.9 (5.3)	26.0 (6.7)	
Code 0	2 (2.7%)	0 (0%)	2 (2.7%)	
Code 1	18 (24.0%)	4 (5.3%)	22 (29.3%)	
Code 2	16 (21.3%)	31 (41.3%)	47 (62.7%)	
Code 3	0 (0%)	4 (5.3%)	4 (5.3%)	

The most frequently prescribed medication was metformin in 51.4% and the average time since diagnosis of diabetes mellitus was 56 months (37) Table 2.

**Table 2:** Duration and Management of diabetics (include frequency of medication and duration of diagnosis)

Drug and frequency of medication (%)	Metformin (51.4)	Glipizide (30.8)	Glucophage (17.8)
Duration of diagnosis (months)	Mean = 56	Minimum = 2	Maximum = 144

The mean value for CRP in diabetics was 0.05 ± 0.04 µg/ml, which was significantly higher than for healthy controls 0.02 ± 0.02 μg/ml (p value < 0.0001). The mean TNF-α value for diabetics was 5.39 ± 12.10 pg/ml, which was also significantly higher than ‘for healthy controls 1.51 ± 3.66 pg/ml, (p = 0.0357) which was significant (Table 3).

**Table 3:** Salivary levels of CRP, IL-6, TNF-α and HbA1C in Type 2 Diabetics and healthy controls

Salivary biomarkers	Mean	Standard deviation	Median	Interquartile range	Test Statistics	p-value
				(Q_3_–Q_1_)		
CRP (μg/ml)						
Controls	0.02	0.02	0.01	(0.021–0.001) = 0.02		
Diabetics	0.05	0.04	0.04	(0.062–0.023) = 0.04	− 4.938*	< 0.0001
IL-6 (pg/ml)						
Controls	41.94	16.88	48	(53.50–26.0) = 27.5	− 1.2828**	0.2036
Diabetics	47.2	18.49	50	(60.0–28.0) = 32.0		
TNF α (pg/ml)						
Controls	1.51	3.66	0.13	(0.215–0.108) = 0.11	− 2.100*	0.0357
Diabetics	5.39	12.1	0.2	(3.661–0.122) = 3.54		
HbA1c (%)						
Controls	4.79	0.65	4.6	(5.10–4.35) = 0.75	− 6.327*	< 0.0001
Diabetics	7.34	2.02	6.9	(9.40–5.60) = 3.80		

^*^Mann–Whitney U test

^**^Paired t test

The mean serum HbA1C levels of diabetics (7.34 ± 2.02%) was significantly higher than the mean serum HbA1C levels in healthy controls (4.79 ± 0.65%), p < 0.0001 (Table 3).

In the multivariate analysis adjusting for age and periodontal status, only the mean salivary CRP was significantly higher in diabetics, 0.034 higher than controls (95% CI 0.009, 0.059 and p = 0.01). The differences in IL-6 and TNF-α levels in diabetics and controls were not significantly different after adjusting for age and periodontal status (Table 4).

**Table 4:** Salivary levels of CRP, IL-6 and TNF-α in type 2 diabetics using healthy controls as reference adjusting for age and periodontal status

Salivary marker	Coefficient	Standard error	95% CI	p value
CRP (μg/ml)	0.0339879	0.0127632	0.008526, 0.0594498	0.010
IL-6 (pg/ml)	3.559502	6.707975	− 9.822543, 16.94155	0.597
TNF α (pg/ml)	− 1.099039	3.312051	− 7.706401, 5.508322	0.741

**Fig. 1:**
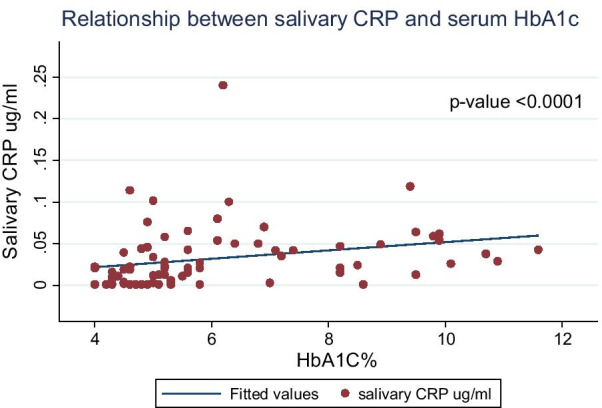
Relationship between salivary CRP and serum HbA1C levels in type 2 diabetics

There was a positive correlation between all the salivary inflammatory biomarkers and HbA1C in Type 2 diabetics (Table 5). However, it was only the relationship between CRP and HbA1C with a correlation coefficient of 0.4929 (p < 0.001) that attained statistical significance in type 2 diabetics. IL-6 and TNF-α were not significant (Fig. 1).

**Table 5:** Spearman correlation between salivary inflammatory biomarkers and serum glycated haemoglobin in type 2 diabetics

	Serum HbA1c	Salivary TNFα	Salivary IL-6	Salivary CRP	p-value
Salivary CRP r-value	0.4929*	0.0946	0.1471	1	< 0.0001
Salivary IL-6 r-value	0.1908	0.0546	1	0.1471	0.1011
Salivary TNFα r-value	0.0041	1	0.0546	0.0946	0.9724
Serum HbA1C r-value	1	0.0041	0.1908	0.4929*	

Spearman correlation *p value < 0.05

## Discussion

Type 2 diabetes mellitus represents a significant global health problem [13]. Currently, diagnosis of diabetes mellitus is made by invasively assessing serological parameters which may be quite discomforting to the patient [7]. Interestingly, novel methods of diagnosis are recently being employed which involve using non-invasive methods to obtain specimens such as saliva [6].

This study showed that the CRP, IL-6, TNF-α levels in saliva were higher in diabetics than healthy controls, supporting the hypothesis that the pathogenesis of type 2 diabetes mellitus involves subclinical chronic inflammation [14, 15]. The mean salivary CRP level in this study was significantly higher in diabetics than in healthy controls. This is in agreement with studies which showed that salivary levels of CRP is higher in diabetics than healthy controls [6, 16]. High salivary CRP levels indicate a low grade inflammatory process that accompanies types 2 diabetes mellitus and has exhibited a highly significant diagnostic capability [16]. Similarly, the mean salivary TNF-α level in this study was higher in diabetics than healthy controls. This is similar to the observation by other authors [17] and [18]. A study reported that the salivary TNF–α levels were significantly higher in subjects with type 2 diabetes mellitus when compared with healthy controls suggesting that diabetes mellitus is associated with increased salivary TNF-α levels [17]. Another study showed that the levels of TNF-α in saliva were significantly increased in subjects with type 2 diabetes mellitus compared with healthy controls [18]. Inflammatory cytokines such as TNF-α are increased in chronic conditions such as type 2 diabetes mellitus and play a role in insulin resistance and has been suggested as an intermediary link between obesity and inflammatory diseases, including type 2 diabetes mellitus and heart disease [19].

The salivary levels of IL-6 were higher in diabetics than healthy controls, but the difference was not significant. This finding contrasts with the work done by other researchers [6], [18] which showed that the salivary levels of IL-6 were significantly higher in subjects with type 2 diabetes mellitus than healthy controls. The variance in their findings compared with this study could be due to difference in the methodology. In the study conducted by [6], unstimulated whole saliva was collected by drooling method and stored in a protease inhibitor cocktail after it has been centrifuged at 4000 rpm for 10 min, the saliva sample was also depleted of all amylase and immunoglobulin by incubating serially with antihuman amylase and protein G beads, before the levels of IL-6 was determined using ELISA, while Monea et al. used the sandwich ELISA method to assay for the levels of IL-6 in unstimulated whole saliva [18]. In our study, unstimulated whole saliva was collected using spit method, centrifuged at 6000 rpm for 10 min to remove contaminants, the supernatant was analysed by ELISA and the levels of IL-6 was determined. However, the major difference in the methodologies are the methods of saliva collection and the storage medium used, this could result in the difference in the results observed among the studies. IL-6 has been demonstrated to be a candidate biomarker for early detection of type 2 diabetes mellitus because it promotes hepatic secretion of triglyceride, which has been suggested as a link between inflammation and the pathogenesis of type 2 diabetes mellitus [20].

We also showed a positive correlation between all the salivary inflammatory biomarkers and serum HbA1C levels in type 2 diabetics. However, the correlation between salivary CRP and serum HbA1C levels was the strongest with correlation coefficient (r) of 0.4929 with p value < 0.0001. Insulin resistance is a central feature in the natural history of type 2 diabetes mellitus, of which CRP play a crucial role [21, 22]. IL-6 has both pro-inflammatory and anti-inflammatory actions [23, 24]. IL-6 also triggers hepatic synthesis of CRP, while TNF-α is more involved in the activation of inflammatory cytokine, endothelial dysfunction and induction of bone resorption [25]. Furthermore, our findings agree with Monea et al., where there was a positive correlation between salivary TNF-α, IL-6 and serum HbA1C levels but the correlations were not statistically significant. The r values reported by Monea et al. for TNF − α and IL-6 were 0.106 and 0.504 respectively while the p value for TNF-α and IL-6 were 0.708 and 0.057. Correlation between salivary CRP and HbA1C was however, not done in their study [18]. Our findings revealed that salivary CRP levels increased with increasing serum HbA1C levels, suggesting an association between glycaemic control and systemic inflammation in participants with established type 2 diabetes. However, there is a paucity of literature on the correlation of salivary CRP levels with HbA1C levels in type 2 diabetics. The positive association of salivary CRP and HbA1C suggests that inflammation is the main hallmark of insulin resistance which is observed in type 2 diabetes mellitus [26]. Low grade systemic inflammation lead to early activation of the immune system with supplementary increase in serum CRP and HbA1c in prediabetes leading to frank diabetes [27]. Other conditions such as periodontal diseases, cardiac inflammation, obesity and renal dysfunction may also be associated with raised CRP levels this is a limitation for the use of these biomarkers for monitoring in diabetes mellitus [28]. Scientific reports have shown a strong association between periodontitis and diabetes, Actinomycosis actinomycetemcomitans (Aa), a facultative gram negative organism play a critical role in pathogenesis of periodontitis, high serum levels of immunoglobulin –A and Immunoglobulin G antibodies against Aa have been detected in the early stage of diabetes [29, 30]. Indeed, periodontal treatment in diabetics led to a reduction in hsCRP after a follow up period [31]. Expectedly, the serum glycated haemoglobin levels were significantly higher in diabetics than healthy controls in our study. This is consistent with a previous Nigerian study that showed the mean HbA1C among diabetics was 7.9 ± 2.4% and was statistically higher than in healthy controls 4.5 ± 1.8%, p < 0.001 [32]. Previous estimates of HbA1C in type 2 diabetics from our institution showed a mean of 8.83 ± 0.42% [33]. Similarly, a review of more than 12 years of using HbA1C levels in the monitoring of glycaemic control among patients in Nigerian hospitals revealed that the mean glycated haemoglobin ranged from 7.9 ± 2.4% to 8.3 ± 2.2% [34].

The present study has some limitations. One of which was that the relationship between salivary and serum (IL-6, TNF-α and CRP) was not investigated, also, the longitudinal relationship between age and salivary (IL-6, TNF-α and CRP) was not explored.

In conclusion, our study showed an association between salivary inflammatory biomarkers (IL-6, CRP, and TNF − α) levels and glycaemic control in participants with type 2 diabetes and suggest that salivary CRP has potential as a non-invasive diagnostic and monitoring tool to assess the glycaemic status of diabetics. Further studies with larger populations that also take confounders that could increase salivary inflammatory markers such as the periodontal status are needed to verify the role of these biomarkers.

## Data Availability

The datasets used and/or analysed during the current study are available from the corresponding author on reasonable request.

## Abbreviations

IL-6: Interleukin 6
TNF-α: Tumour necrosis factor
CRP: C- reactive protein
HbA1c: Glycated haemoglobin
BPE: Basic periodontal examination
OHI-S: Simplified oral hygiene index
PD: Pocket depth
ELISA: Enzyme linked immunosorbent assay.
Aa: Actinomycetes acetinomycetmcomitans
